# Home Blood Pressure Telemonitoring Technology for Patients With Asymptomatic Elevated Blood Pressure Discharged From the Emergency Department: Pilot Study

**DOI:** 10.2196/49592

**Published:** 2024-01-30

**Authors:** Karen C Tran, Meagan Mak, Laura M Kuyper, Jesse Bittman, Birinder Mangat, Heather Lindsay, Chad Kim Sing, Liang Xu, Hubert Wong, Martin Dawes, Nadia Khan, Kendall Ho

**Affiliations:** 1 Division of General Internal Medicine Faculty of Medicine University of British Columbia Vancouver, BC Canada; 2 Center for Health Evaluation and Outcome Sciences Vancouver, BC Canada; 3 Department of Emergency Medicine Faculty of Medicine University of British Columbia Vancouver, BC Canada; 4 Division of Family Practice Faculty of Medicine University of British Columbia Vancouver, BC Canada

**Keywords:** hypertension, remote-home monitoring, feasibility study, health monitor, telehealth, pilot study, mobile phone, monitoring, telemonitoring, blood pressure, emergency department, hypertension, morbidity, mortality, primary care, physician care, management, hypertension medication

## Abstract

**Background:**

Hypertension affects 1 in 5 Canadians and is the leading cause of morbidity and mortality globally. Hypertension control is declining due to multiple factors including lack of access to primary care. Consequently, patients with hypertension frequently visit the emergency department (ED) due to high blood pressure (BP). Telehealth for Emergency-Community Continuity of Care Connectivity via Home-Telemonitoring Blood Pressure is a pilot project that implements and evaluates a comprehensive home blood pressure telemonitoring (HBPT) and physician case management protocol designed as a postdischarge management strategy to support patients with asymptomatic elevated BP as they transition from the ED to home.

**Objective:**

Our objective was to conduct a feasibility study of an HBPT program for patients with asymptomatic elevated BP discharged from the ED.

**Methods:**

Patients discharged from an urban, tertiary care hospital ED with asymptomatic elevated BP were recruited in Vancouver, British Columbia, Canada, and provided with HBPT technology for 3 months of monitoring post discharge and referred to specialist hypertension clinics. Participants monitored their BP twice in the morning and evenings and tele-transmitted readings via Bluetooth Sensor each day using an app. A monitoring clinician received these data and monitored the patient’s condition daily and adjusted antihypertensive medications. Feasibility outcomes included eligibility, recruitment, adherence to monitoring, and retention rates. Secondary outcomes included proportion of those who were defined as having hypertension post-ED visits, changes in mean BP, overall BP control, medication adherence, changes to antihypertensive medications, quality of life, and end user experience at 3 months.

**Results:**

A total of 46 multiethnic patients (mean age 63, SD 17 years, 69%, n=32 women) found to have severe hypertension (mean 191, SD 23/mean 100, SD 14 mm Hg) in the ED were recruited, initiated on HBPT with hypertension specialist physician referral and followed up for 3 months. Eligibility and recruitment rates were 40% (56/139) and 88% (49/56), respectively. The proportion of participants that completed ≥80% of home BP measurements at 1 and 3 months were 67% (31/46) and 41% (19/46), respectively. The proportion of individuals who achieved home systolic BP and diastolic BP control at 3 months was 71.4% (30/42) and 85.7% (36/42) respectively. Mean home systolic and diastolic BP improved by –13/–5 mm Hg after initiation of HBPT to the end of the study. Patients were prescribed 1 additional antihypertensive medication. No differences in medication adherence from enrollment to 3 months were noted. Most patients (76%, 25/33) were highly satisfied with the HBPT program and 76% (25/33) found digital health tools easy to use.

**Conclusions:**

HBPT intervention is a feasible postdischarge management strategy and can be beneficial in supporting patients with asymptomatic elevated BP from the ED. A randomized trial is underway to evaluate the efficacy of this intervention on BP control.

## Introduction

Hypertension is the leading cause of death and disability worldwide [1,2]. Long-term poor control of blood pressure (BP) can result in significant cardiovascular (CV) morbidity and mortality [3]. Severely asymptomatic elevated BP in the emergency department (ED) has recently been associated with undiagnosed hypertension and adverse CV outcomes [4,5], regardless of the initial reason (eg, pain or anxiety) for the ED visit [6]. In a meta-analysis of 12 studies (n=1240) of individuals with elevated BP in ED, 43.4% were diagnosed with hypertension at follow-up [7]. Among individuals discharged from ED with elevated BP, two-thirds still had uncontrolled BP at 6 months [8].

Hypertension is one of the few chronic conditions that can be monitored online, with home blood pressure telemonitoring (HBPT), which allows tele-transmission of BP in real time to central health portal, eliminating the need for in-person BP visits between patients and health care providers. Importantly, close monitoring of BP and data can be summarized by patients and providers, including calculating of BP averages, graphing temporal BP, and flagging high or low values [9,10]. This is particularly important for patients with asymptomatic elevated BP presenting to and discharged from the ED. Current ED guidelines recommends no routine ED management and outpatient follow-up without suggesting any additional monitoring or intervention with a few exceptions [11,12]. As these patients transition from acute to community settings, their BP can remain dangerously elevated or lowered with initiation of antihypertensive medications. Hypertension telemedicine studies have been shown to be highly feasible, effective, and acceptable to patients [13-17]. Although a promising intervention, to date, no studies have leveraged HBPT as a postdischarge management strategy for patients discharged from the ED with asymptomatic elevated BP.

Telehealth for Emergency-Community Continuity of Care Connectivity via Home-Telemonitoring Blood Pressure (TEC4Home-BP) is a pilot study to evaluate the feasibility of HBPT and physician case management as an integrated component of health delivery to support patients with asymptomatic elevated BP discharged from the ED to home. The primary objective of this pilot study is to report the feasibility of HBPT as a postdischarge management strategy and the secondary objective is to determine acceptability of this initiative. We hypothesize that the study procedures would be feasible and acceptable to patients.

## Methods

### Recruitment Procedures

The feasibility pilot study was an unblinded trial. Participants were prospectively recruited from 1 large academic urban ED in Vancouver, British Columbia, Canada, from May to December 2021. We included adults (older than 19 years of age) who presented to the ED with asymptomatic elevated BP confirmed at ED triage and average of 3 subsequent measurements performed in the ED (systolic blood pressure [SBP] ≥ 160 mm Hg or diastolic blood pressure [DBP] ≥ 100 mm Hg), who owned or had daily access to a smartphone, and agreeable to follow-up at the Vancouver General Hospital (VGH) Hypertension Clinic and tele-transmit home BP readings via the Sphygmo app. Individuals with 1 of the following conditions were excluded, patients with hypertensive emergencies with evidence of end organ involvement, stroke or acute coronary syndrome, people who are pregnant, acute intoxication, acute surgical or trauma, patients who are psychiatrically unstable, advanced cognitive impairment, patients requiring admission to hospital, inability to use or care for home BP monitor correctly, from long-term care facility, unstable housing, and are non–English-speaking with no family members who can help translate.

Participants were recruited through 2 recruitment pathways. Potential participants were first identified via referrals from hospital ED staff and were screened by a research assistant in hospital ED (recruitment pathway 1). ED staffs were asked to refer the study team any patients presenting to the ED with suspected hypertension, patients whose BP was above SBP ≥ 160 mm Hg or DBP ≥ 100 mm Hg and in stable condition. Once the research assistant was notified of the patient referrals, the research assistant reviewed their medical records and excluded any patients who met exclusion criteria from screening. Only patients who appear to meet all inclusion criteria and remain potentially eligible were approached for further screening and have subsequent BP readings measured to confirm their eligibility. During the recruitment, the study team expanded referral streams to include patients with asymptomatic elevated BP who were referred to the VGH Hypertension Clinic following discharge from the ED to increase recruitment numbers due to limits on research activities in the ED during the COVID-19 pandemic (recruitment pathway 2). Physicians at the Hypertension Clinic screened all incoming referrals based on inclusion and exclusion criteria, and selected eligible patients for further contact. Eligible patients were contacted either in ED, immediately after discharge, or once the referral to Hypertension Clinic was received. Participants completed the consent process and were enrolled within 7 days from ED discharge.

### Ethical Considerations

Ethics approval was obtained from the University of British Columbia Research Ethics Board (#H20-03207). The written informed consent was obtained from all participants. All data collected from the participants are de-identified and remain anonymous. A privacy impact assessment was completed to ensure the telemonitoring application is compliant with all University of British Columbia and Vancouver Coastal Health privacy policies. Participants were allowed to keep the BP telemonitor device at the end of the monitoring period as a gift for participating in the study.

### HBPT and Hypertension Clinic Intervention

All patients were provided with a validated electronic upper arm oscillometric BP telemonitor device (A&D Ltd UA-641BLE) with wireless data transfer (Bluetooth) capabilities using a smartphone [18,19]. The research assistant assisted the patients to set up the Sphygmo app on their smartphones, and connected the BP telemonitor device to their smartphones via Bluetooth. Patients were then taught to follow the on-screen instructions in the app to complete and submit their BP readings, properly measure their BP, and view their current and previous BP readings. They were instructed to perform home BP monitoring schedules recommended by Hypertension Canada and International Society of Hypertension Virtual Management of hypertension guidelines, consisting of duplicate measurements in the morning and evening for 7 consecutive days [20,21]. For each 7-day HBPT series, the first day’s measurements were discarded and the mean of subsequent measurements calculated and used to guide medication titration. Home BP readings were transmitted and telemonitored via Sphygmo app on a smartphone, which is PIPEDA (Personal Information Protection and Electronic Documents Act)-, PIPA (Personal Information Protection Act)-, and HIPAA (Health Insurance Portability and Accountability Act)-compliant, with encryption on both ends and a medical server based in Ontario, Canada. Tele-transmitted home BP readings were monitored daily by a monitoring clinician, who would contact participants by telephone if BP was uncontrolled (SBP ≥ 180, DBP ≥ 100 mm Hg or SBP ≤ 100 mm Hg) according to BP management algorithm (Multimedia Appendix 1). Urgency of hypertension clinic follow-up was dependent on severity of telemonitored home BP readings post-ED discharge (Multimedia Appendix 1).

Patients were seen at the VGH Hypertension Clinic either in person or phone assessment. Physicians assessed patients using standardized hypertension intake forms, administered behavioral counseling, encouraged medication adherence, reviewed telemonitored BP summaries, adjusted BP medications accordingly, and arranged clinical follow-up as needed based on Hypertension Canada guidelines [20]. At minimum, patients were seen at the time of enrollment for initial consultation, and at 1 and 3 months for follow-up. Target home BP values were defined by Hypertension Canada of <135/85 mm Hg or <130/80 mm Hg for those with diabetes [20]. At the end of the study, a summary of participant’s home BP readings were sent to their primary care provider.

### Data Collection and Outcomes

#### Overview

Baseline data including sociodemographic, ethnicity, education level, health behaviors, and relevant medical history were collected. Antihypertensive medication history was reconciled and number of antihypertensive medications, class of antihypertensive medications, and hypertensive defined daily dose (HDDD) were recorded according to patient self-report at enrollment and at 3 months (Multimedia Appendix 2). HDDD quantitatively describes the intensity of a patient’s overall antihypertensive medication regimen [22]. Medication adherences were assessed using the validated Hill Bone Medication Adherence Scale (HB-MAS) at the time of enrollment and at 3 months [23]. Health-related quality of life (QoL) was assessed using EQ-5D-5L at the end of the study [24]. Satisfaction surveys were sent to participants for completion at the end of 3 months via REDCap (Research Electronic Data Capture; Vanderbilt University; Multimedia Appendix 3). A total of 3 reminder emails were sent to participants to complete the surveys.

Feasibility outcomes were eligibility, recruitment, retention rate, and adherence rate. Eligibility rate was defined as proportion of participants who were deemed eligible to participate among all the patients that were screened. Recruitment rate was defined as the proportion of participants who are deemed eligible and who consented to participate in the study. Retention rate was defined as the proportion of the participants that completed 1 week of HBPT and attended first Hypertension Clinic visit. Home monitoring adherence was defined as the percentage of participants that completed 80% of HBPT at 1- and 3-month follow-up visits.

Secondary outcomes included the proportion of the participants meeting the definition of hypertension at 3 months (defined as mean home SBP ≥ 135 mm Hg or DBP ≥ 85 mm Hg or antihypertensive prescription), mean change in HBPM from enrollment to 1- and 3-month follow-ups, and proportion of participations meeting home BP targets (defined as mean home BP readings of <135/85 mm Hg or <130/80 mm Hg if having diabetes) at 3 months. Additional outcomes of interest were number of antihypertensive medications, HDDD, medication adherence (HB-MAS), health-related QoL (EQ-5D), and patient satisfaction questionnaires.

#### Statistical Analysis

Characteristics of included participants were described as mean, SD, median, IQR, and proportions. BP changes and changes in other study outcomes (baseline to 3 months) were summarized using means and SDs or median and IQR. For participants who had BP recordings within a window from 14 days before to 14 days after the target 90-day follow-up date, the 3-month BP was taken to be the average the BP recordings of up to 3 days closest to the target date. For participants who did not have BP recordings within this window, but who had data over at least 60 days, multiple imputation was used to assign the 3-month BP (see Multimedia Appendix 4 for details). The remaining participants were excluded from the analysis of BP change as it was deemed not possible to assign them reliable 3-month BP values. Patients who completed either 3-month questionnaire were included in the analysis and descriptive statistics were used. Data were analyzed using Stata (StataCorp) and R (version 4.1.2; R Core Team).

## Results

### Recruitment

**Figure 1:**
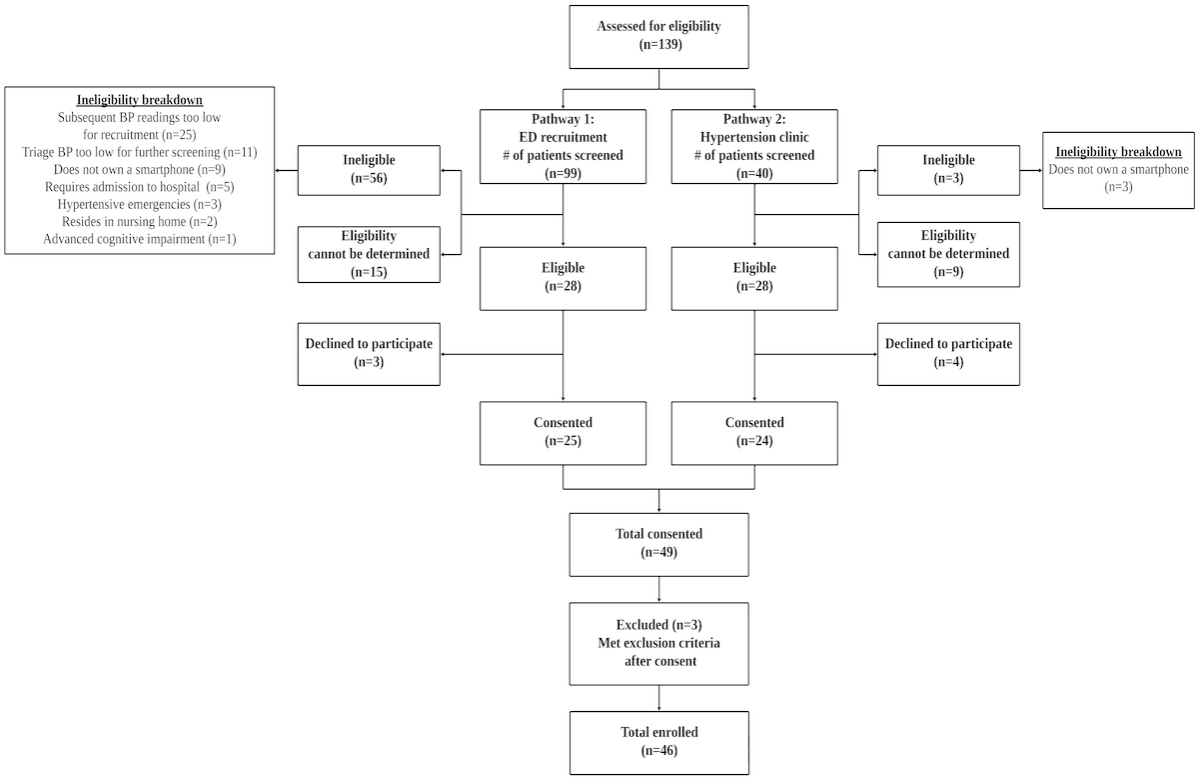
Flow diagram of patient participant screening and recruitment. BP: blood pressure; ED: emergency department.

**Figure 2:**
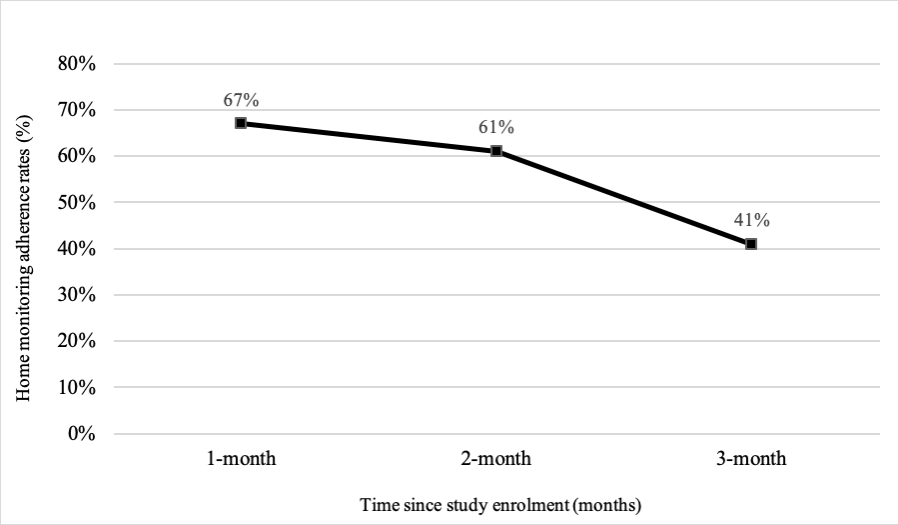
Home monitoring adherence rates at 1, 2, and 3 months of home blood pressure telemonitoring (n=46).

From May to December 2021, a total of 139 patients presenting to the ED with asymptomatic elevated BP were screened, including 99 identified in ED (recruitment pathway 1) and 40 identified from ED referrals to Hypertension Clinic (recruitment pathway 2). Potentially eligible patients that were referred to the research team by ED staff had an average triage BP of 192/94 and were contacted by the research assistant for additional BP measurements. Among the patients who had additional BP measured by the research assistant, those who were ineligible had an average BP of 147/81 (n=26), whereas eligible patients had an average BP of 194/99 (n=32). Including patients identified and screened through ED referrals to the Hypertension Clinic (recruitment pathway 2), 56 patients were eligible to be consented to the study (Figure 1), and 49 were enrolled. Eligibility and recruitment rates were 40% (56/139) and 88% (49/56), respectively. After enrollment, 3 participants were deemed ineligible and were excluded from the study due to requiring hospitalization (n=3). The final analyzed cohort consisted of 46 participants. Of which, 44/46 patients completed at least 1 week of HBPT and 1 clinic visit at the Hypertension Clinic, resulting in a retention rate of 98% (44/66). Home monitoring adherence was defined as the percentage of participants that completed 80% of the prespecified home blood pressure monitoring schedule at 1 and 3 months from enrollment. The proportion of participants that completed ≥80% of home BP measurements at 1 and 3 months were 67% (31/46) and 41% (19/46), respectively (Figure 2). A total of 4 patients did not have sufficient home BP readings at the end of 3 months to determine mean home BP and were lost to follow-up.

### Characteristics of Participants

Participants were mostly women (69%, n=32) with an average age of 63 (SD 17) years (Table 1). The ethnic diversity of participants was White (30%, n=14), Filipino (22%, n=10), and Chinese (15%, n=7). At baseline, the mean (SD) SBP and DBP in the ED were 193 (SD 23) mm Hg and 100 (SD 13) mm Hg, respectively. Of all the participants, 44 (96%) had been previously diagnosed with hypertension. Of all participants, 17 (37%) had an antihypertensive medication initiated or increased in the ED. Those who had an intensification of medications had higher mean BP in ED of 205/104 mm Hg compared to those who did not of 186/98 mm Hg.

**Table 1:** Baseline demographics of enrolled participants.

Baseline		All participants (N=46)
**Sociodemographic**		
	Age (years), mean (SD)	63 (17)
	Women, n (%)	32 (69)
**Race and ethnicity, n (%)**		
	White	14 (30)
	Filipino	10 (22)
	Chinese	7 (15)
	South Asian	4 (9)
	Indigenous	3 (7)
	Korean	1 (2)
	Southeast Asian	1 (2)
	Other	3 (7)
	Preferred not to answer	1 (2)
	Did not answer	2 (4)
**Health behavior, n (%)**		
	Current smoker	1 (2)
**Education level, n (%)**		
	University degree above bachelor’s level	3 (7)
	University degree at bachelor’s level	15 (33)
	University certificate below bachelor’s level	4 (9)
	Trade certificate or diploma from a vocational school or apprenticeship training	2 (4)
	Nonuniversity certificate or diploma from a community college	6 (13)
	High school graduation	11 (24)
	Less than high school graduation	2 (4)
	Preferred not to answer	1 (2)
	Did not answer	2 (4)
**Medical history, n (%)**		
	Hypertension	44 (96)
	Diabetes	9 (20)
	Dyslipidemia	21 (46)
	Coronary artery disease	4 (9)
	Heart failure	1 (2)
	Atrial fibrillation	2 (4)
	Stroke	4 (9)
	Chronic kidney disease	2 (4)
	Erectile dysfunction	1 (2)
	Vascular dementia	1 (2)
**Antihypertensive medications**		
	Taking antihypertensive medication, n (%)	10 (22)
	Number of antihypertensive medication, mean (SD)	1.58 (1.32)
**Clinical measures,** **mean (SD)**		
	Framingham risk score	14.8 (3.9)
	HB-MAS^a^	36 (3)
	EQ-5D-5L index^b^ value	0.765 (0.23)
	EQ-5D-5L VAS^c^	68.88 (19.91)
**Blood pressure in emergency department,** **mean (SD)**		
	Systolic BP^d^	193 (23)
	Diastolic BP	100 (14)

^a^HB-MAS: Hill Bone Medication Adherence Scale.

^b^Index value calculated from 5 domains: mobility, self-care, usual activities, pain or discomfort, and anxiety or depression. Value calculated using cross walk tool, range 0.3-1; higher scores are better quality of life [24].

^c^VAS: Visual Analogue Scale. Range 0-100; higher scores are better quality of life [24].

^d^BP: blood pressure.

### Patient Outcomes and Experience

**Figure 3:**
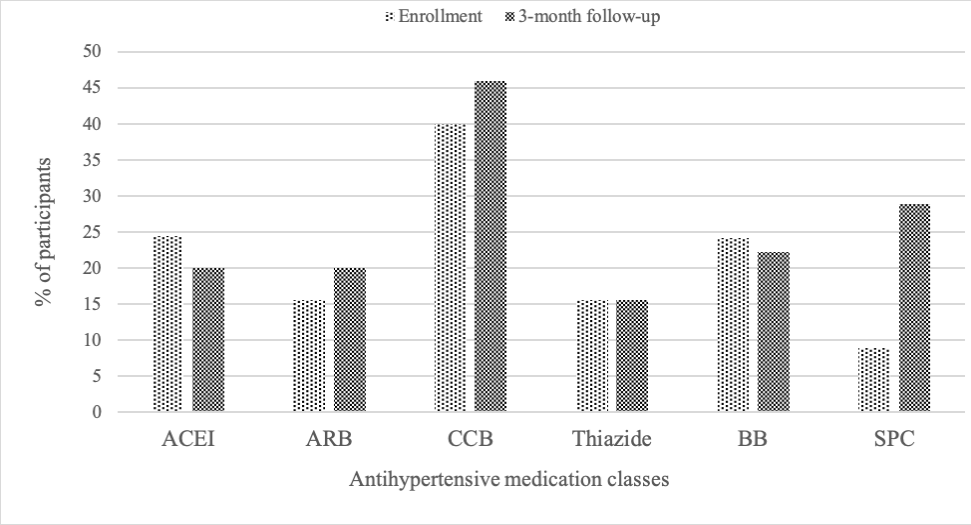
Change in distribution of antihypertensive medication classes from study enrollment to 3-month follow-up in a group of participants with elevated blood pressure (n=46). ACEI: angiotensin-converting enzyme inhibitor; ARB: angiotensin receptor blocker; BB: beta-blocker; CCB: calcium channel blocker; SPC: single pill combination.

There were sufficient HBPT data to determine the 3-month BP end point for 42 (91%) participants, with imputation used for 1 participant. Mean home SBP and DBP decreased by 67.0 (SD 24.4) and 24.9 (SD 10.3), respectively, from ED triage or screening to study completion. Similarly, mean home SBP and DBP decreased by 13.2 (SD 17.8) mm Hg and 5.1 (SD 9.0) mm Hg, respectively, from the first 7 days to study completion. The proportion of individuals who achieved home SBP and DBP control at 3 months was 71% (30/42) and 86% (36/42), respectively (n=42). The number of adjustments in participant medications was 87 for the entire study, with most commonly having initiation of new antihypertensive medication (36/87), increase in dosage of current antihypertensive medication (23/87), and change in class of antihypertensive medication (14/87). Decreasing antihypertensive medications (9/87) and stopping antihypertensive medications (5/87) were uncommon. At the end of the study, patients were prescribed 1 additional antihypertensive medication (2 vs 1 antihypertensive medication), but no difference in HDDD was noted from enrollment to end of the study (mean 1.58, SD 1.32 vs mean 1.77, SD 1.47; *P*=.39; Table 2). Most commonly patients at the beginning of the study were taking angiotensin-converting enzyme inhibitors or angiotensin receptor blockers (40%, 18/45), calcium channel blockers (40%, 18/45), and beta-blockers (24%, 11/45). At the end of the intervention, more patients were prescribed single pill combination antihypertensive medications (9%, 4/45 vs 29%, 13/45; Figure 3).

The response rate for completion of both the HB-MAS at enrollment and the end of the study was 72% (33/46). Among those who completed both questionnaires, no difference in medication adherence was noted (36 [IQR 36-33] vs 36 [IQR 36-35]) from enrollment to 3 months (Table 2). The response rate for completion of the EQ-5D validated questionnaire was 97% (44/46; Table 1). EQ-5D-5L and EQ-5D Visual Analogue Scale (EQ-5D VAS) scores were 0.77 (SD 0.23) and 69 (SD 20), respectively. Due to the pilot nature of the study, adverse events were not captured.

The response rate for the patient satisfaction surveys was 72% (33/46). Overall, patients were highly satisfied with the HBPT program (75%, 24/33) and would recommend it to others (79%, 26/33; Table 3). The majority of the participants found digital health tools easy to use (76%, 25/33) and felt that the intervention prevented the need to return to the ED with elevated BP readings (64%, 21/33). In total, 14% (7/46) of participants required family assistance to participate in the study. The most common reasons for assistance were language barrier (71%, 5/7), inability to apply the home BP monitor on their arm independently (14%, 1/7), and lack of their own smartphone (14%, 1/7). Patients who did not have their own smartphones navigated this barrier by using their family members’ phones.

**Table 2:** Change in blood pressure and additional secondary outcomes.

Participants	Enrollment (N=46)	First week of HBPT^a^ (N=46)	3-month F/U^b^ (N=42)	Change at 3-month F/U (from enrollment; N=42)	*P* value	Change at 3-month F/U (from first week of HBPT; N=42)
SBP^c^, mean (SD)	193 (23)	140 (16)	127 (12)	–66 (24)	N/A^d^	–13 (17)
DBP^e^, mean (SD)	100 (13)	81 (12)	75 (9)	–25 (10)	N/A	–5 (9)
Number of antihypertensive medications, mean (SD)	1.58 (1.32)	1.58 (1.32)	1.77 (1.47)	N/A	N/A	N/A
HDDD^f^, mean (SD)	1.49 (1.22)	1.58 (1.32)	1.77 (1.47)	N/A	.39	N/A
HB-MAS^g^ (IQR), mean (SD)	36 (3)	36 (3)	36 (2)	0.66 (2)	N/A	N/A

^a^HBPT: home blood pressure telemonitoring.

^b^F/U: follow-up.

^c^SBP: systolic blood pressure.

^d^N/A: not applicable.

^e^DBP: diastolic blood pressure.

^f^HDDD: hypertensive daily defined dose.

^g^HB-MAS: Hill Bone Medication Adherence Scale.

**Table 3:** The proportion of participants (n=33) that selected “highly satisfied” or “strongly agree” to questions on the participant satisfaction questionnaire following completion of the study.

Survey question	Selected highly satisfied and strongly agree, n (%)
Overall satisfaction	24 (75)
Satisfaction with health care coaching	26 (79)
Would recommend to other patients	26 (79)
Easy to use	25 (76)
Better management of health conditions	24 (73)
Satisfaction with progress toward health goals	22 (67)
More informed about chronic conditions	21 (64)
Less need to visit ED^a^	21 (64)
Improvement in QoL^b^	17 (52)
Family satisfied with care provided	17 (52)
Less need to visit family doctor	16 (49)

^a^ED: emergency department.

^b^QoL: quality of life.

## Discussion

### Overview

TEC4Home-BP is a pilot study to evaluate the feasibility of HBPT and physician case management as an integrated component of health delivery to support patients with asymptomatic elevated BP discharged from the ED to home. We found that HBPT was feasible as a postdischarge management strategy, given our recruitment and retention rate was 88% (56/139) and 98% (44/46), respectively. Patients reported high acceptability and satisfaction with the HBPT program.

### Principal Results

HBPT combined with timely physician follow-up and management is a novel and promising postdischarge management strategy to help bridge the transition from ED to home and can play a proactive role in treating asymptomatic elevated BP in the ED. Our results show that the proportion achieving home SBP and DBP control at 3 months was 71% (30/42) and 86% (36/42), respectively, which is higher than the current Canadian hypertension control rates of 65% [25]. Furthermore, home BP decreased from the first week of completing HBPT to 3 months (final BP assessment) by –13/–5 mm Hg. This may correspond to an estimated 30% and 26% risk reduction in CV disease and stroke, respectively, but likely overestimated due to the limitations of our study design with regression of the mean and lack of a comparator group [26]. Overall, patients only required 1 additional antihypertensive medication to be prescribed. Improvement in BP may be due to increased use of evidence-based medications, including single pill combination therapies or the act of self-monitoring. Patients were highly satisfied with the program and found the technology to be user-friendly.

### Comparison With Prior Work

Previous studies have shown that text-messaging services to encourage measuring home BP using a wrist cuff was a feasible intervention in patients who were discharged from the ED with high BP and reduced SBP by 9.1 (95% CI 1.1-17.6) mm Hg, but patients did not use remote HBPT program where home BP readings were transmitted to physicians to review and provide management strategies directly to patients [27]. Our results showed that the adherence to home BP monitoring was 67% (31/46) and 41% (19/46) at 1 and 3 months, respectively. This is similar to meta-analysis showing that among 13 studies (n=1662 patients), the average adherence to telemedicine-based hypertension management was high (76.8%; range 48%-90%) [28]. This is not surprising that as time persists from their initial ED event, patients may not be as adherent to strict BP monitoring. It will be important for our future randomized controlled trial (RCT) to develop a more pragmatic home BP monitoring schedule that is acceptable to patients and provide accurate information to health care professionals to monitor and manage their hypertension.

Previous studies from ED and inpatient hospital settings have shown that in patients discharged with asymptomatic elevated BP, 43.4% were diagnosed with hypertension at follow-up [7]. Our study showed that among individuals presenting the ED with an average SBP ≥ 160 mm Hg and DBP ≥ 100 mm Hg in ED, most had established hypertension that was uncontrolled and required additional treatment. This reaffirms that elevated BP in the ED is not benign and a significant proportion of these individuals have undiagnosed or undertreated hypertension. Of similar importance, is the portion of patients that do not go on to have a diagnosis of hypertension and are at risk of misdiagnosis and unnecessary treatment without close follow-up. Furthermore, with worsening hypertension awareness and control rates in Canada, the ED may be a useful location to screen individuals for hypertension, as BP is measured in all individuals regardless of the presenting complaint. Despite the guideline recommendations that close follow-up is needed for individuals with asymptomatic elevated BP discharged from ED [12], only 7%-29% of patients with elevated BP receive any hypertension assessment, treatment, or referral in the ED [29-32]. Therefore, the development of novel postdischarge management pathways to ensure that these individuals have a close follow-up for their hypertension is greatly needed. At a minimum, ED practice guidelines should consider changing to at least recommend home BP monitoring for these patients immediately postdischarge and timely follow-up with physicians should BP remain elevated.

Our intervention improved BP after 1 week of HBPT to 3 months (final BP assessment) by –13/–5 mm Hg. Meta-analyses have shown that home BP monitoring supported by cointerventions (including medication adjustments by physicians or pharmacists, education, and lifestyle counseling) results in significant BP reduction (–6.1, 95% CI –9.0 to –3.2 mm Hg) that persists for 12 months [16]. Another meta-analysis of 23 RCTs (n=7037 patients) reported that HBPT significantly reduced BP by 5/3 mm Hg compared to the usual care (*P*<.001) [17]. Our results need to be verified by conducting a powered RCT to address issues with regression to the mean and lack of a comparator group.

Studies have shown that HBPT alone improved antihypertensive medication prescription and QoL [17,33], but we were not able to show this in our pilot study as it was not powered to detect these differences. Although there were no differences in HDDD prescribed, there was an increase in the number of single pill combination antihypertensive medications prescribed. We hypothesize that HDDD did not change because single pill combinations were more frequently prescribed which confers better BP lowering with combination therapy than full doses of antihypertensive medication [34]. Hypertension follow-up after an ED visit for asymptomatic elevated BP has been shown to improve evidence-based hypertension management [35]. Single pill combinations are endorsed by hypertension guidelines [20] as they have been shown to improve medication adherence and BP control while reducing medication side effects and CV events [36-39].

Our intervention was noted to be highly acceptable and usable by patients and their families, which is similar to other HBPT studies where patients reported higher satisfaction and greater convenience compared with usual hypertension care [40]. Almost 15% (7/46) of participants required assistance with our intervention, specifically due to language barriers. Given our multiethnic population, future RCT design should incorporate different languages in the technology to improve usability for everyone.

### Limitations

There are a number of limitations in this study. The sample size for the TEC4Home-BP pilot study was relatively small and there may be systematic differences between those who enrolled for the study and adhered to the monitoring protocol and those who did not. Although the retention rate for participants attending the first visit was high, adherence to the monitoring schedule declined throughout the study and 4 patients were lost to follow-up. This insight on monitoring frequency has provided us with information on how to redesign on monitoring protocol to improve patient adherence. There is increasing evidence that a minimum of 12 BP readings is needed for home BP measurements to be valid [41], and more recently 3 days of BP readings appeared sufficient to prognostic home BP readings [42]. Furthermore, the exclusion criteria excluded many patients who presented to the ED with elevated BP and only stable patients who would be a good candidate for HBPT were recruited for the study. This limitation prevents our feasibility study from assessing the feasibility of HBPT in the full group of patients who present to the ED with elevated BP, and who may still benefit from and accept HBPT. The eligibility criteria may be underestimated, as we did not document all the reasons why patients were not eligible to be in the study due to resource issues. A majority of patients who had elevated triage BPs were not eligible to participate because their BP was below the inclusion criteria after the research assistant measured their BP 3 times consecutively. In addition, those who did not meet the incomplete survey questionnaires may limit the true acceptability and usability of this intervention, as participants who responded and did not respond may be very different. Finally, the lack of data from a control comparison is a true limitation of our feasibility study design and needs to be addressed. Although, these findings are promising and informative of the next steps, a more rigorous RCT design with a comparator group is required to test the true efficacy of this intervention.

### Conclusions

HBPT with physician management is a feasible and acceptable postdischarge management strategy to monitor patients with asymptomatic elevated BP when they are discharged from the ED. Future multicenter RCTs are needed to evaluate the efficacy of this intervention in a large population.

## Appendix

Multimedia Appendix 1 Physician decision management tool.

Multimedia Appendix 2 Hypertension defined daily dose.

Multimedia Appendix 3 Participant survey.

Multimedia Appendix 4 Statistical methods.

## Abbreviations

BP: blood pressure
CV: cardiovascular
DBP: diastolic blood pressure
ED: emergency department
EQ-5D VAS: EQ-5D Visual Analogue Scale
HB-MAS: Hill Bone Medication Adherence Scale
HBPT: home blood pressure telemonitoring
HDDD: hypertensive defined daily dose
HIPAA: Health Insurance Portability and Accountability Act
PIPA: Personal Information Protection Act
PIPEDA: Personal Information Protection and Electronic Documents Act
QoL: quality of life
RCT: randomized controlled trial
REDCap: Research Electronic Data Capture
SBP: systolic blood pressure
TEC4Home-BP: Telehealth for Emergency-Community Continuity of Care Connectivity via Home-Telemonitoring Blood Pressure
VGH: Vancouver General Hospital

## Data Availability

The data sets generated and analyzed during this study are available from the corresponding author on reasonable request.
