# Supplementary material for: Home Blood Pressure Telemonitoring Technology for Patients With Asymptomatic Elevated Blood Pressure Discharged From the Emergency Department: Pilot Study
Source: JMIR Form Res. 2024 Jan 30;8:e49592. doi: 10.2196/49592 (PMC10865197; doi:10.2196/49592)
Supplement: Multimedia Appendix 1 [file formative_v8i1e49592_app1.docx]

**TEC4Home BP Decision Support Tool: Timing of First Initial Consultation**

Continue HHM Service

Continue HHM Service

Continue HHM Service

Book follow up within **1 week**

Book follow up in **48 hours**

Book follow up within **2 weeks**

Book follow up in **48 hours**

Book follow up within **2-4 weeks**

IF SBP 135-149 or DBP 86-90

IF SBP < 100

IF SBP 150-179 or DBP > 90-99

Continue HHM Service

Continue HHM Service

Review HHM trends for 3-5 days on Sphygmo App

Review paper and electronic record

NO

YES

IF SBP ≥ 180 or DBP ≥ 100

IF SBP 100-135 or DBP ≤ 85

Any data triggering an alert

**TEC4Home BP Decision Support Tool: Ongoing Monitoring and Follow up**

Contact patient **within 48 hours** to confirm medication adherence, consider changes to medications after discussing with MD, and arrange follow up in 1 week

≥

Contact patient **within 2 weeks** to confirm medication adherence, consider changes to medications, after discussing with MD

≥

Contact patient **within 48 hours** to confirm medication adherence, consider changes to medications after discussing with MD, and arrange follow up in 1 week

≥

NO

Contact patient **within 2 weeks** to confirm medication adherence

IF SBP 135-149 or DBP 86-90

Continue HHM Service

IF SBP < 100

Continue HHM Service

IF SBP 150-179 or DBP 90-99

Continue HHM Service

Continue HHM Service

Review HHM trends for past week on Sphygmo App

Review paper and electronic record

Continue HHM Service

YES

IF SBP ≥ 180 or DBP ≥ 100

IF SBP 100-135 or DBP ≤ 85

Any data triggering an alert
