# Supplementary material for: Home Blood Pressure Telemonitoring Technology for Patients With Asymptomatic Elevated Blood Pressure Discharged From the Emergency Department: Pilot Study
Source: JMIR Form Res. 2024 Jan 30;8:e49592. doi: 10.2196/49592 (PMC10865197; doi:10.2196/49592)
Supplement: Multimedia Appendix 2 [file formative_v8i1e49592_app2.docx]

**APPENDIX**

**Hypertensive Defined Daily Doses (HDDD) Used in TEC4Home BP Analysis**

| **Class** | **Drug** | **Min et al’s**  **HDDD** | **Canadian Max dose** | **Recommended Daily Maintenance Doses** | **HDDD used in Analysis** |
| --- | --- | --- | --- | --- | --- |
| **Angiotensin Converting Enzyme Inhibitor (ACEI)** | Benazepril | 20 | 40 | 20 | **20** |
|  | Captopril | 50 | 150 | 75 | **50** |
|  | Cilazapril | NA | 10 | 5 | **5** |
|  | Enalapril | 3 | 40 | 5 | **5** |
|  | Fosinopril | 20 | 40 | 20 | **20** |
|  | Lisinopril | 20 | 40 | 20 | **20** |
|  | Perindopril | 8 | 8 | 4 | **4** |
|  | Quinapril | 40 | 40 | 20 | **20** |
|  | Ramipril | 10 | 20 | 10 | **10** |
|  | Trandolapril | 2 | 4 | 2 | **2** |
| **Alpha Adrenergic Blocker** | Doxazosin | N/A | 16 | 8 | **8** |
|  | Prazosin | N/A | 20 | 10 | **10** |
|  | Terazosin | N/A | 20 | 5 | **5** |
| **Angiotensin Receptor Blocker (ARB)** | Azilsartan | N/A | 80 | 40 | **40** |
|  | Candesartan | 16 | 32 | 8 | **32** |
|  | Eprosartan | 600 | 600 | 600 | **600** |
|  | Irbesartan | 150 | 300 | 150 | **150** |
|  | Losartan | 50 | 100 | 50 | **50** |
|  | Olmesartan | 20 | 40 | 20 | **20** |
|  | Telmisartan | 40 | 80 | 80 | **80** |
|  | Valsartan | 160 | 320 | 160 | **80** |
| **Beta-Adrenergic Antagonists (Beta-Blockers)** | Acebutolol | 400 | 800 | 400 | **200** |
|  | Atenolol | 50 | 100 | 50 | **50** |
|  | Bisoprolol | 5 | 20 | 10 | **5** |
|  | Carvedilol | 25 | 50 |  | **25** |
|  | Labetalol | 400 | 1200 | 400 | **200** |
|  | Metoprolol | 100 | 400 | 200 | **100** |
|  | Nadolol | 80 | 320 | 160 | **160** |
|  | Nebivolol | 20 | 20 | 10 | **10** |
|  | Pindolol | 30 | 60 | 30 | **30** |
|  | Propanolol | 80 | 480 | 240 | **80** |
|  | Timolol | N/A | 60 | 20 | **20** |
| **Centrally acting agents** | Clonidine | 0.4 | 0.6 | 0.6 | **0.6** |
|  | Methyldopa | 500 | 3000 | 2000 | **500** |
| **Calcium Channel Blocker (dihydropyridine)** | Amlodipine | 5 | 10 | 5 | **5** |
|  | Felodipine | 5 | 20 | 10 | **5** |
|  | Nifedipine | 60 | 120 | 60 | **60** |
| **Direct Renin Inhibitor** | Aliskiren | 150 | 300 | 150 | **150** |
| **Calcium Channel Blocker (non-dihydropyridine)** | Diltiazem | 240 | 360 | 240 | **240** |
|  | Verapamil | 240 | 480 | 240 | **240** |
| **Potassium sparing diuretic** | Amiloride | 5 | 40 | 10 | **10** |
|  | Spironolactone | 50 | 200 | 100 | **50** |
| **Thiazide/**  **Thiazide-like Diuretic** | Chlorthalidone | 12.5 | 50 | 25 | **25** |
|  | Hydrochlorothiazide | 25 | 50 | 25 | **25** |
|  | Indapamide | 2.5 | 5 | 2.5 | **2.5** |
|  | Metolazone | 5 | 10 | 5 | **5** |
| **Vasodilator** | Hydralazine | 100 | 300 | 200 | **200** |
|  | Minoxidil | 20 | 100 | 20 | **20** |
| **Combination Product** | | | | | **Single dose per day** |
