# Supplementary material for: Home Blood Pressure Telemonitoring Technology for Patients With Asymptomatic Elevated Blood Pressure Discharged From the Emergency Department: Pilot Study
Source: JMIR Form Res. 2024 Jan 30;8:e49592. doi: 10.2196/49592 (PMC10865197; doi:10.2196/49592)
Supplement: Multimedia Appendix 3 [file formative_v8i1e49592_app3.docx]

**SYSTEM AND USE SURVEY (Tec4Home BP PATIENT)^^[[1]](#footnote-1)^^**

*This survey is about the home BP tele-monitoring system your health care provider uses to monitor your health and blood pressure remotely (i.e, from your home).*

1. In general, **how satisfied are you overall** with the TEC4Home BP Monitoring program in which you are currently enrolled?

| Highly Satisfied | Moderately satisfied | Neither satisfied nor dissatisfied | Moderately dissatisfied | Not at all satisfied |
| --- | --- | --- | --- | --- |
|  |  |  |  |  |

1. **How satisfied are you with the quality** of the health care coaching provided to you by the TEC4Home BP program?

| Highly Satisfied | Moderately satisfied | Neither satisfied nor dissatisfied | Moderately dissatisfied | Not at all satisfied |
| --- | --- | --- | --- | --- |
|  |  |  |  |  |

1. **How satisfied are you with your progress towards the health goals set out at the beginning of the TEC4Home BP program?**

| Highly Satisfied | Moderately satisfied | Neither satisfied nor dissatisfied | Moderately dissatisfied | Not at all satisfied |
| --- | --- | --- | --- | --- |
|  |  |  |  |  |

1. Please indicate your **level of agreement or disagreement** with each of the following statements below:

|  | Strongly  Agree | Moderately  Agree | Moderately  Disagree | Strongly  Disagree | Not  Sure | N/A |
| --- | --- | --- | --- | --- | --- | --- |
| 1. Participating in this program has **improved my quality of life**. |  |  |  |  |  |  |
| 1. Participating in this program allows me to **better manage my own health condition**. |  |  |  |  |  |  |
| 1. Participating in this program means my family and/or caregiver(s) feel confident that I am **getting the care I need** |  |  |  |  |  |  |
|  |  |  |  |  |  |  |
|  | Strongly Agree | Moderately Agree | Moderately Disagree | Strongly Disagree | Not Sure | N/A |
| 1. I feel **more informed about my chronic condition** as a result of participating in this program. |  |  |  |  |  |  |
| 1. I have had **less need to visit an Emergency Department** since starting the program. |  |  |  |  |  |  |
| 1. I have had **less need to visit my family doctor/GP** or walk-in clinic since starting the program. |  |  |  |  |  |  |
| 1. The digital health tools/connected devices used in the program are **easy to use**. *(if applicable)* |  |  |  |  |  |  |
| 1. I feel the program **adequately provides for the privacy and security** of my information |  |  |  |  |  |  |

1. The TEC4Home BP Home Health Monitoring program **has saved me time** by not having to travel to see a health care provider.

| Strongly Agree | Moderately Agree | Moderately Disagree | Strongly Disagree | Not Sure | N/A |
| --- | --- | --- | --- | --- | --- |
|  |  |  |  |  |  |

If Strongly or Moderately Agree, how much **time on average** **did it save me** over the course of one month? _____________ hours

1. The TEC4Home BP Home Health Monitoring program **has saved my family member(s) or caregiver time** by not needing to travel to assist me or take me to appointments.

| Strongly Agree | Moderately Agree | Moderately Disagree | Strongly Disagree | Not Sure | N/A |
| --- | --- | --- | --- | --- | --- |
|  |  |  |  |  |  |

If Strongly or Moderately Agree, how much **time on average did it save them** over the course of one month? _____________ hours

1. Thinking of the last time that you experienced a medical problem related to your condition for which you’re enrolled in the program, **what did you do**? (Check all that apply)

| Used the **Telehomecare program** to communicate with care providers | Had a **telephone visit** with my regular health care provider | Had an **in-person visit** with my regular health care provider. | Visited a **walk-in clinic** | Visited an **emergency department** | **Did not seek care** | **Other (specify)** |
| --- | --- | --- | --- | --- | --- | --- |
|  |  |  |  |  |  |  |

1. Prior to enrolling in the TEC4Home BP Home Health Monitoring program, **what would you have most likely done**? (Check all that apply)

| Had a **telephone visit** with my regular health care provider | Had an **in-person visit** with my regular health care provider. | Visited a **walk-in clinic** | Visited an **emergency department** | **Would not seek care** | **Other (specify)** |
| --- | --- | --- | --- | --- | --- |
|  |  |  |  |  |  |

1. How **likely** are you to recommend TEC4Home Home Health Monitoring program to other patients with similar medical problems?

| **Definitely** | **Probably** | **May or May Not** | **Probably Not** | **Definitely Not** |
| --- | --- | --- | --- | --- |
|  |  |  |  |  |

1. Do you have **any other comments** you would like to make regarding this program?

**ADDITIONAL QUESTIONS**

1. What were your expectations when you signed up for the study and started the TEC4Home BP monitoring program? Were your expectations met, or did they change during your participation?
   _________________________________________________________________________________

_________________________________________________________________________________

_________________________________________________________________________________

_________________________________________________________________________________

1. Did you receive enough information, training, and support to use the monitoring equipment?
   _________________________________________________________________________________

_________________________________________________________________________________

_________________________________________________________________________________

_________________________________________________________________________________

1. Is there anything you know now that you would have liked to have known when you started?
   _________________________________________________________________________________

_________________________________________________________________________________

_________________________________________________________________________________

_________________________________________________________________________________

1. Do you feel that TEC4Home BP and the monitoring experience changed the way you understand your high blood pressure? If so, how?
   _________________________________________________________________________________

_________________________________________________________________________________

_________________________________________________________________________________

_________________________________________________________________________________

1. Regarding your experiences participating with TEC4Home BP or any aspects of the project, is there anything that we did not already discuss that you would like to mention?
   _________________________________________________________________________________

_________________________________________________________________________________

_________________________________________________________________________________

_________________________________________________________________________________

**Thank you for taking the time to share your experiences and knowledge!**

**We value your input. Please ensure to return this survey.**

1. Canada Health Infoway © 2017 [↑](#footnote-ref-1)
