# Supplementary material for: Home Blood Pressure Telemonitoring Technology for Patients With Asymptomatic Elevated Blood Pressure Discharged From the Emergency Department: Pilot Study
Source: JMIR Form Res. 2024 Jan 30;8:e49592. doi: 10.2196/49592 (PMC10865197; doi:10.2196/49592)
Supplement: Multimedia Appendix 4 [file formative_v8i1e49592_app4.docx]

*Details on imputation*

The 3-months BP was imputed for participants with at least 60 days of recordings. For each of systolic and diastolic BP, the average of recordings for each day was computed and used for imputation. The imputation model was a simple regression model with time as the sole covariate. The R package ‘mice’ (version 3.15.0) was used for calculations. The number of multiple imputations was set to 10 and the method was set to Bayesian linear regression.
